# Supplementary material for: Artificial Liver Support with CytoSorb and MARS in Liver Failure: A Retrospective Propensity Matched Analysis
Source: J Clin Med. 2023 Mar 14;12(6):2258. doi: 10.3390/jcm12062258 (PMC10058971; doi:10.3390/jcm12062258)
Supplement: Supplementary file 1 [file jcm-12-02258-s001.zip › jcm-2248748-supplementary.pdf]

Supplementary Table S1. Comparison on clinical and paraclinical data and severity scores at ICU admission and ICU discharge.

	CytoSorb group			MARS group			P between groups
	ICU admission	ICU discharge	P value	ICU admission	ICU discharge	P value	
Lactate (µmol/L)	2.9 [1.2, 7.8]	3.1 [0.7, 9.2]	0.95	2.4 [1.1, 8.2]	8.9 [1.2, 25.0]	0.02	0.01
Creatinine (mg/dL)	0.8 [0.2, 6.0]	1.3 [0.5, 4.9]	0.44	0.6 [0.1, 2.4]	1.4 [0.3, 6.2]	0.01	0.23
BUN (mg/dL)	60 [3, 276]	57 [9, 294]	0.91	30 [8, 132]	31 [11, 184]	0.76	0.50
Bilirubin (mg/dL)	19.4 [4.0, 33.8]	10.1 [0.6, 33.8]	0.14	25.3 [3.2, 51.0]	11.6 [0.7, 42.6]	0.11	0.54
Sodium (mmol/L)	138 [119, 141]	137 [123, 147]	0.58	137 [126, 143]	138 [131, 141]	0.23	0.74
INR	2.7 [1.5, 11.2]	2.0 [1.0, 5.6]	0.07	2.6 [1.6, 5.7]	2.3 [1.1. 17.3]	0.54	0.18
Albumin (g/dL)	2.7 [1.7, 3.7]	3.2 [1.8, 4.2]	0.17	3.2 [2.2, 4.4]	2.9 [1.9, 5.4]	0.57	0.33
AST (U/L)	98 [46, 8219]	65 [23, 1164]	0.12	249 [50, 8219]	103 [23, 312]	0.01	0.56
ALT (U/L)	257 [31, 8332]	73 [9, 832]	0.04	257 [7, 7550]	89 [18, 552]	0.05	0.71
GGT (U/L)	63 [28, 489]	88 [15, 774]	0.85	70 [16, 512]	29 [14, 99]	0.01	0.31

Ammonia (μmol/L/)	69 [26, 147]	39 [2, 168]	0.16	29 [15, 110]	93 [21, 123]	0.10	0.03
LDH (U/L)	418 [290, 1832]	273 [104, 1817]	0.05	302 [132, 3679]	313 [163, 555]	0.97	0.35
Haemoglobin (g/dL)	10.6 [6.7, 15.6]	7.8 [6.9, 10.6]	0.02	10.8 [5.7, 15.2]	7.5 [6.0, 10.5]	0.01	0.77
WBC (*10^3/μL)	13.6 [6.8, 34.9]	11.9 [1.9, 23.1]	0.57	10.6 [2.8, 18.2]	8.7 [0.6, 81.6]	0.94	0.18
Platelets (*10^3/μL)	111 [43, 303]	37 [20, 244]	0.02	107 [28, 262]	42 [6.0, 397]	0.01	0.26
PCT (ng/mL)	0.9 [0.1, 5.8]	0.5 [0.1, 13.9]	0.39	1.0 [0.1, 2.5]	0.8 [0.2, 2.1]	0.72	0.54
PCR (mg/L)	12.0 [0.5, 137.0]	32.0 [1.9, 115.0]	0.05	15.2 [2.5, 51.8]	29 [2.7, 56.0]	0.58	0.03
Fibrinogen (mg/dL)	145 [83, 387]	157 [50, 382]	0.95	128 [77, 286]	124 [27, 409]	0.40	0.58
SOFA Score	9 [5, 13]	13 [1, 21]	0.20	7 [4, 14]	14 [1, 22]	0.03	0.93
SOFA CV	0 [0, 1]	4 [0, 4]	0.01	0 [0, 3]	2 [0, 4]	0.02	0.79
SOFA Resp	1 [0, 3]	2 [0, 3]	0.82	2 [0, 3]	2 [0, 4]	0.65	0.73
SOFA Coag	1 [0, 3]	3 [0, 3]	0.01	1 [0, 3]	3 [0, 4]	0.03	0.76
SOFA Liver	4 [2, 4]	3 [0, 4]	0.12	4 [2, 4]	4 [0, 4]	0.25	0.92
SOFA Renal	0 [0, 4]	3 [0, 4]	0.02	0 [0, 3]	1 [0, 4]	0.03	0.30
SOFA CNS	1 [1, 4]	2 [0, 4]	0.84	0 [0, 4]	4 [0, 4]	0.08	0.23

MELD Score	34 [24, 40]	36 [6, 40]	0.80	32 [18, 40]	29 [9, 40]	0.80	0.50
GCS (points)	13 [3, 14]	13 [3, 15]	0.13	15 [3, 15]	13 [3, 15]	0.11	0.86
MAP (mmHg)	72 [68, 98]	70 [65, 91]	0.96	90 [69, 122]	78 [60, 96]	0.01	0.01
HR (bpm)	83 [65, 125]	90 [63, 133]	0.87	83 [56, 116]	104 [48, 114]	0.20	0.47

Legend. Data are presented as median [min, max]. BUN, urea; INR, International Normalized Ratio; AST, aspartate aminotransferase; ALT, alanine aminotransferase; GGT, gamma-glutamyl transferase; LDH, lactate dehydrogenase; WBC, white blood cell count; CRP, C-reactive protein; PCT, procalcitonin, SOFA, Sequential Organ Failure Assessment; CV, cardiovascular; Resp, respiratory; Coag, coagulation; CNS, Central Nervous System; GCS, Glasgow Coma Scale; MAP, mean arterial pressure; HR, heart rate
